# Parametric versus non-parametric statistics in the analysis of randomized trials with non-normally distributed data

**DOI:** 10.1186/1471-2288-5-35

**Published:** 2005-11-03

**Authors:** Andrew J Vickers

**Affiliations:** 1 Integrative Medicine Service, Biostatistics Service, Memorial Sloan Kettering Cancer Center, Howard 1312a, 1275 York Avenue, NY, NY 10021, USA

## Abstract

**Background:**

It has generally been argued that parametric statistics should not be applied to data with non-normal distributions. Empirical research has demonstrated that Mann-Whitney generally has greater power than the *t*-test unless data are sampled from the normal. In the case of randomized trials, we are typically interested in how an endpoint, such as blood pressure or pain, changes following treatment. Such trials should be analyzed using ANCOVA, rather than *t*-test. The objectives of this study were: a) to compare the relative power of Mann-Whitney and ANCOVA; b) to determine whether ANCOVA provides an unbiased estimate for the difference between groups; c) to investigate the distribution of change scores between repeat assessments of a non-normally distributed variable.

**Methods:**

Polynomials were developed to simulate five archetypal non-normal distributions for baseline and post-treatment scores in a randomized trial. Simulation studies compared the power of Mann-Whitney and ANCOVA for analyzing each distribution, varying sample size, correlation and type of treatment effect (ratio or shift).

**Results:**

Change between skewed baseline and post-treatment data tended towards a normal distribution. ANCOVA was generally superior to Mann-Whitney in most situations, especially where log-transformed data were entered into the model. The estimate of the treatment effect from ANCOVA was not importantly biased.

**Conclusion:**

ANCOVA is the preferred method of analyzing randomized trials with baseline and post-treatment measures. In certain extreme cases, ANCOVA is less powerful than Mann-Whitney. Notably, in these cases, the estimate of treatment effect provided by ANCOVA is of questionable interpretability.

## Background

Introductory statistics textbooks typically advise against the use of parametric methods, such as the *t*-test, for the analysis of randomized trials unless data approximate to a normal distribution. Altman, for example, states that "parametric methods require the observations within each group to have an approximately Normal distribution ... if the raw data do not satisfy these conditions ... a non-parametric method should be used" [1]. In some cases, central limit theorem is invoked such that parametric methods are said to be applicable if sample size is suitably large: "for reasonably large samples (say, 30 or more observations in each sample) ... the *t*-test may be computed on almost any set of continuous data" [2].

The rationale for recommending non-parametric over parametric methods, unless certain conditions are met, is rarely made explicit. But techniques for statistical inference from randomized trials can only fail in one of two ways: they can inappropriately reject the null hypothesis of no difference between groups (false positive or Type I error) or inappropriately fail to reject the null (false negative or Type II error). Hence any recommendation to favor one technique over another must be based on the relative rates of these two errors.

Empirical statistical research has clearly demonstrated that the *t*-test does not inflate Type I (false positive) error. In a typical study, Heeren et al examined the properties of the *t*-test to analyze small two-group trials where data are ordinal, such as from a five point scale, and thus non-normal [3]. They found that where there was truly no difference between groups, the *t*-test would reject the null hypothesis close to 5% of the time.

Thus concern over the relative advantages of parametric and non-parametric methods has focussed on Type II error [4]. Typically, researchers have created a large number of data sets, in which observations were created from a distribution incorporating a difference between groups. Each data set is then analyzed by both parametric and non-parametric methods in order to calculate the proportion of times the null hypothesis is rejected (that is, the power) [5-7].

The results have been fairly consistent. Where data are sampled from a normal distribution, the *t*-test has very slightly higher power than Mann-Whitney, the non-parametric alternative. However, when data are sampled from any one of a variety of non-normal distributions, Mann-Whitney is superior, often by a large amount. Bridge and Sawilowsky, for example, concluded that" "the *t*-test was more powerful only under a distribution that was relatively symmetric, although the magnitude of the differences was trivial. In contrast, the [Mann-Whitney] held huge power advantages for data sets which presented skewness" [7]. Many workers have linked results showing the superiority of non-parametric methods for non-normal distributions to claims that data rarely follow a normal distribution (as Micceri puts it: "The unicorn, the normal curve and other improbable creatures" [8]). This has led to implicit recommendations that non-parametric techniques should be considered the method of choice [7].

It is arguable, however, that these prior investigations are flawed. The *t*-test and Mann-Whitney are used for continuous variables such as blood pressure, depression, weight or pain. Most commonly, we are interested in seeing how these variables *change *following an intervention. This reflects clinical practice where the patient presents with a problem and asks the doctor to help improve it. In a typical study, a patient with hypertension, obesity or chronic headache is randomized to drug or placebo to see whether the drug is effective for reducing blood pressure, weight or pain. The researchers might report that, say, blood pressure fell by 5 mm in the placebo group but by 14 mm in the drug group. Indeed, trials in which we are interested only in post-treatment scores, and where change is not of interest, are rather rare, being primarily confined to iatrogenic symptoms such as post-operative pain or chemotherapy vomiting.

**Figure 1:**
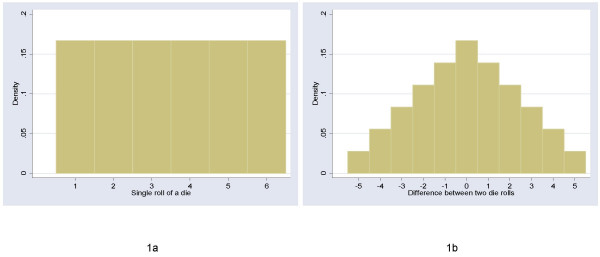
Distribution of scores for a single die roll and the difference between two die rolls. The change score tends towards a more normal distribution.

There are two implications for methodologic research on the relative value of parametric and non-parametric techniques. First, we should worry about the distribution of change scores. It seems likely that change from baseline would approximate more closely to a normal distribution than the post-treatment score. This is because change scores are a linear combination and the Central Limit Theorem therefore applies. As a simple example, imagine that baseline and post-treatment score were represented by a single throw of a die. The post-treatment score has a flat (uniform) distribution, with each possible value having an equal probability (figure 1a). The change score has a more normal distribution: there is a peak in the middle at zero – the chance of a zero change score is the same as the chance of throwing the same number twice, that is 1 in 6 – with more rare events at the extremes – there is only a 1 in 18 chance of increasing or decreasing score by 5 (Figure 1b).

Moreover, where an endpoint is measured at baseline and again at follow-up, the *t*-test is not the recommended parametric method. Analysis of covariance (ANCOVA), where baseline score is added as a covariate in a linear regression, has been shown to be more powerful than the *t*-test [9-11]. It has several additional advantages: it adjusts for any chance baseline imbalances; it can be extended to incorporate randomization strata as co-variates, which has been shown to increase power [12]; it can also be extended to incorporate time effects where measures are repeated.

In this paper, I report results from a study making the more rational comparison between parametric and non-parametric methods: ANCOVA and Mann-Whitney. Such a comparison does not appear to have been reported previously. I aimed to compare relative power of the two methods under a variety of distributions. As a secondary objective, I aimed to determine whether ANCOVA provided an unbiased estimate for the difference between groups where data did not follow a normal distribution. A third, overarching aim was to investigate the distribution of change scores between repeat assessments of a non-normally distributed variable.

## Methods

**Figure 2:**
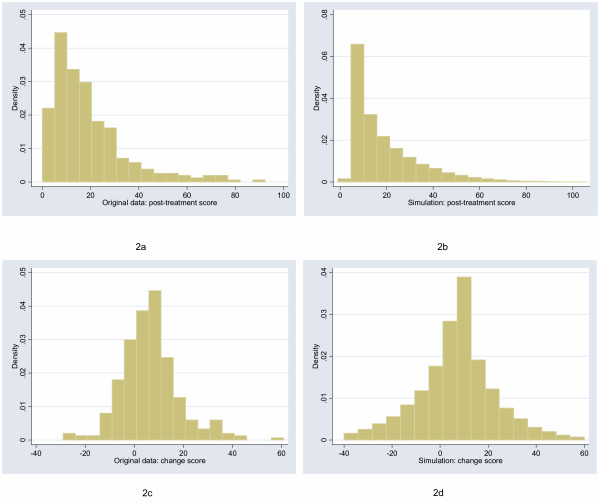
Distribution of post-treatment and change scores from original and simulated data for headache severity ("moderate positive skew" distribution).

**Figure 3:**
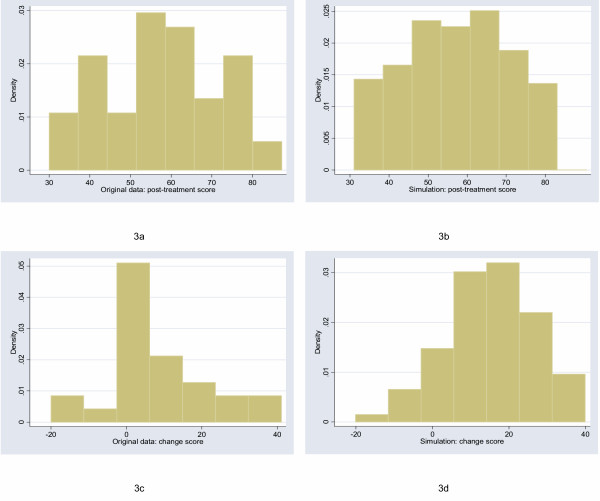
Distribution of post-treatment and change scores from original and simulated data for shoulder pain ("uniform" distribution).

**Figure 4:**
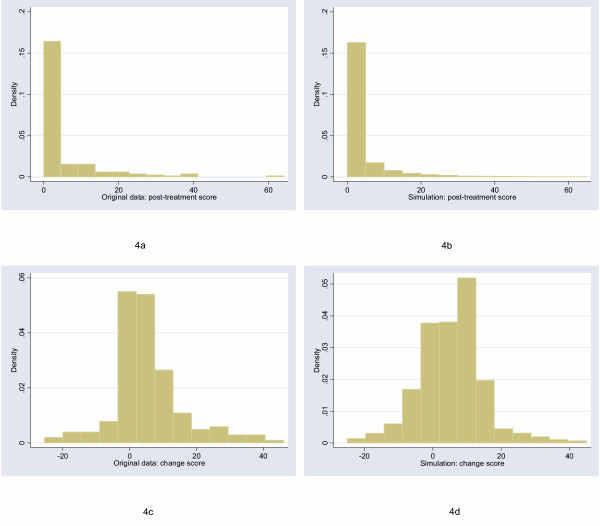
Distribution of post-treatment and change scores from original and simulated data for Ki67, a biomarker of cell proliferation ("extreme asymmetry" distribution).

**Figure 5:**
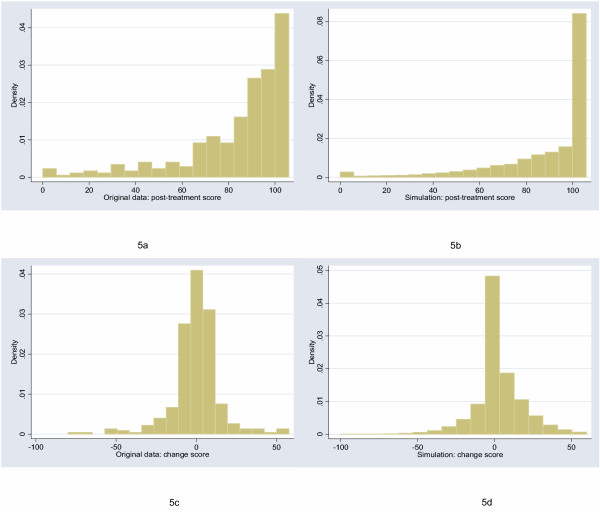
Distribution of post-treatment and change scores from original and simulated data for physical functioning scale of the SF36 ("extreme negative skew" distribution).

The starting point for this study was to obtain archetypal data sets for analysis. I will follow Bridge [7] in choosing empirical rather than theoretical distributions. I examined the distribution of a large number of empirical data sets and cross-referenced these with those described by Micceri, who systematically obtained 440 data sets from the psychological and educational domains [8]. The most common distribution appeared one with moderate positive skew. As an exemplar, I used a headache severity index from a large (n = 401) randomized trial of headache prophylaxis [13] (Figure 2). This distribution was also used with scores reversed, to create a distribution with moderate negative skew. A second pain data set, this time from a trial on athletes with shoulder pain [14], provides an example of a more uniform distribution (Figure 3). Data on Ki67, an antigen that is a marker for cell proliferation, were obtained from a randomized comparison of two hormonal treatments for breast cancer [15]. The distribution for Ki67 is comparable to Micceri's "extreme asymmetry distribution" (Figure 4). For extreme negative skew, I used data from the physical functioning scale of the SF36 (Figure 5), again taken from the headache trial. As a comparison group, data were also drawn from a normal distribution with a mean of 5 and a standard deviation of 1.

For each of the distributions, I created a polynomial that converted normal data to a distribution with an approximately similar shape. For example, the distribution with moderate positive skew in Figure 2 was simulated by sampling *x *from the normal and creating a new variable equal to 14.8+16.5*x*+7.5*x*^2^-1.15*x*^3^, rounded, like the original scale, to the nearest 0.25. The simulation distributions were compared to the empirical distributions by visual inspection and comparison of the standard deviation, skewness and kurtosis.

To run the simulations, a bivariate normal (mean 0, standard deviation 1) with a specified correlation was created for a trial of a given sample size equally divided in two groups. The polynomial was applied and a treatment effect introduced. The treatment effect was one of two forms: a shift, for example, scores in the treatment group were reduced by two points; and a ratio, for example, treatment group scores were reduced by 20%. Results were then analyzed by Mann-Whitney and ANCOVA, with *p*-values obtained by asymptotic approximation for the Mann-Whitney test. In some simulations, *t*-tests and ANCOVA of log-transformed data were applied. The *t*-test and Mann-Whitney used the follow-up score if correlation was less than 0.5 and the change score otherwise. This maximizes the power of these tests [11] and might be seen as favoring unadjusted tests on the grounds that the correlation between baseline and follow-up scores is not known when the protocol for statistical analysis is written. Note that the correlation cited in the results is the correlation between baseline and follow-up in the control group. Some previous workers have used the overall correlation using both groups when investigating the properties of ANCOVA [11]. The difference between these two values was small in the context of our simulations, for example, a correlation of 0.5 in the control group was equivalent to a correlation of 0.476 for both groups combined.

Simulations were repeated 1000 times for each combination of sample size (10, 20, 30, 40, 60, 100, 200, 400, 800) and correlation (0.1, 0.2, 0.3 ... 0.9) using Stata 8.2 (Stata Corp., College Station, Texas). The exception was extreme asymmetry data for the Ki67 biomarker. The baseline and post-treatment distributions had quite different shapes and different polynomials were used to model each. This constrained the range of possible correlations, hence only the empirical correlation observed in the original study was used, 0.4, with 5000 iterations.

Results were compared between different methods using the "relative efficiency" (RE) measure. This gives the relative number of patients required for a study analyzed using parametric methods so that power was equivalent to the non-parametric alternative. Hence an RE of 1.25 indicates that a particular trial analyzed by parametric statistics would have to accrue 25% more patients than if it were to be analyzed non-parametrically; an AE of 0.80 would indicate that the parametric method was superior by an equivalent amount. The RE is calculated from observed power of the tests, that is, the proportion of simulations in which the *p*-value was less than the α of 5%. Where (1-β_np_) and (1-β_p_) are the observed powers from the simulations for the non-parametric and parametric test respectively, RE is given by the formula:



Note that, although it is arguable that the null hypotheses for different tests, say the *t*-test and Mann-Whitney, are technically different, the conclusions drawn by investigators of a randomized trial given a particular *p*-value will be the same, regardless of the analytic method used. Hence direct comparison of the power of different tests is justified in this setting.

## Results

The figures show the distributions of post-treatment and change scores from the original data and associated simulations. Visual comparison of subfigures (a) with (b), and (c) with (d), suggests that the polynomials used for the simulations produce distributions that are reasonably similar to the related empirical distribution. Comparing subfigures (a) to (c), and (b) with (d), it is apparent that, as hypothesized, the change between baseline and follow-up scores tends towards the normal distribution. These visual impressions are confirmed in Table 1, which shows estimates of the shape parameters for the distributions. The shape parameters for the empirical and simulated data are similar, and skewness is much closer to zero for the change score compared to the follow-up score.

**Table 1:** Shape parameters for the distributions produced by the simulations compared to those from the original empirical data. Parameters for the moderate negative skew are as for the moderate positive skew, except that the sign for skew is reversed.

Distribution	Post-treatment scores					
	
	Standard deviation		Skewness		Kurtosis	
	
	Empirical	Simulation	Empirical	Simulation	Empirical	Simulation
Moderate positive skew	17.01	17.02	1.62	1.63	5.71	6.00
Uniform	13.49	13.49	-0.11	-0.11	2.08	2.01
Extreme asymmetry	8.96	9.78	3.03	2.90	13.77	13.97
Extreme negative skew	22.17	21.88	-1.74	-1.79	5.88	5.85

	Change scores					
	
	Standard deviation		Skewness		Kurtosis	
	
	Empirical	Simulation	Empirical	Simulation	Empirical	Simulation

Moderate positive skew	10.40	18.80	0.35	0.01	4.49	5.15
Uniform	12.31	14.56	0.00	0.43	3.05	2.98
Extreme asymmetry	10.62	10.97	0.88	1.08	5.38	6.37
Extreme negative skew	15.51	15.02	0.00	0.75	6.31	8.52

As a second check on the simulations, Table 2 compares the power of *t*-test and Mann-Whitney. The data for post-treatment scores were obtained by combining all data from simulations where correlation was less than 0.5; the change scores were from data where correlation was 0.5 or more. These results broadly replicate those of previous workers and therefore provide support for the methods of the current study. In particular, the increase in relative efficiency of the *t*-test under normality (or uniform) is trivial compared to its loss in relative power under asymmetry. Two aspects of Table 2 have not been reported previously. First, RE can vary depending on whether the treatment effect is a shift or a ratio change. Second, the power of Mann-Whitney and *t*-test are more similar (RE closer to 1) for change scores, presumably because change scores are more normally distributed. An exception is for extreme asymmetry, where Mann-Whitney has extremely poor power for change scores.

**Table 2:** Relative power of *t*-test and Mann-Whitney given as relative efficiency. Values less than 1 indicate greater power of *t*-test; greater than 1 indicates superiority of Mann-Whitney. Results are combined across sample sizes and correlations.

Distribution	Post-treatment scores	Change scores
Moderate positive skew: shift	0.9348	0.9835
Moderate positive skew: ratio	1.1382	1.0436
Moderate negative skew: shift	1.1833	1.0187
Moderate negative skew: ratio	0.9301	0.9825
Uniform: shift	0.9339	0.9846
Uniform: ratio	0.9488	0.9929
Extreme negative skew: shift	1.3769	1.1140
Extreme negative skew: ratio	1.6675	1.2046
Extreme asymmetry: shift	7.1461	0.5370
Extreme asymmetry: ratio	9.0091	0.6432
Normal: shift	0.9660	0.9726
Normal: ratio	0.9740	0.9760

Table 3 gives RE for each combination of sample size and correlation for the moderate positive skew data, where the treatment effect was a shift. ANCOVA is generally superior to Mann-Whitney. Smaller sample sizes and correlations near the extremes reduce the advantage of ANCOVA. Table 4 shows the RE for each of the different distributions combining data for correlations between 0.4 and 0.7, which constitutes a typical range for correlations described in the literature [16]. Mann-Whitney is superior for some very small sample sizes, but RE is non-trivially larger than 1 across sample sizes only for the extreme negative skew distribution with a ratio treatment effect. In table 5, data are given by correlation, combining sample sizes. The table has one particularly notable feature: for some distributions, RE's drop dramatically between correlation of 0.4 and 0.5. This is apparently because the endpoint analyzed changed from the post-treatment score to the change score at correlations of 0.5 and above. This was to maximize power following previous work on the power of unadjusted tests based on the normal [9,11]. As it seems possible that the relative power of analyzing change and post-treatment scores may differ between the normal and asymmetric case, the data were reanalyzed using post-treatment scores only (see Table 6). In the case of extreme negative skew, the simulation was repeated with ANCOVA on log-transformed data. Cleary, analyzing only post-treatment score, irrespective of correlation, improves the efficiency of Mann-Whitney considerably, but it is still inefficient compared to log-transformed ANCOVA. That said, log-transformed ANCOVA is slightly anti-conservative: when the simulation was repeated with no treatment effect, the null hypothesis was rejected for 5.23% (rather than the nominal 5%) of trials.

**Table 3:** Relative efficiency of ANCOVA and Mann-Whitney for the moderate positive skew data. Values less than 1 indicate greater power of ANCOVA; greater than 1 indicates superiority of Mann-Whitney. In blank cells, the power of one or both tests was 100%.

Sample size	Correlation between baseline and post-treatment score								
	
	0.1	0.2	0.3	0.4	0.5	0.6	0.7	0.8	0.9
10	0.8125	0.9773	0.6017	0.9403	0.9722	0.8834	1.2129	1.0851	1.1320
20	1.0160	1.1093	0.8172	0.9348	0.7742	0.9905	0.8124	1.0058	1.0231
30	1.0000	0.9167	0.7785	0.8617	0.8027	0.7756	0.8689	0.8760	1.0096
40	0.8866	0.8596	0.8120	0.7332	0.7365	0.7556	0.9172	0.9067	0.929
60	0.8925	0.8996	0.7752	0.7632	0.7418	0.8277	0.8728	0.8841	0.9892
100	0.8822	0.8594	0.7816	0.7259	0.7071	0.8277	0.8639	0.8702	0.9259
200	0.8484	0.8030	0.7611	0.6920	0.6979	0.7591	0.8793	0.8888	-
400	0.8512	0.8292	0.7392	0.7113	0.6707	0.8029	0.8336	-	-
800	0.8781	0.9087	-	-	-	-	-	-	-

**Table 4:** Relative efficiency of ANCOVA and Mann-Whitney combining correlations 0.4 – 0.7. Values less than 1 indicate greater power of ANCOVA; greater than 1 indicates superiority of Mann-Whitney. In blank cells, the power of one or both tests was 100%.

Distribution	Sample size								
	
	10	20	30	40	60	100	200	400	800
Moderate positive skew: shift	1.0221	0.8751	0.8292	0.8004	0.8085	0.7887	0.7549	0.7497	-
Moderate positive skew: ratio	1.5001	0.9832	1.0161	0.8441	0.7973	0.8079	0.7755	0.7689	0.8389
Moderate negative skew: shift	1.0045	0.9793	0.8080	0.8300	0.8088	0.7810	0.7772	0.7494	0.7404
Moderate negative skew: ratio	1.7878	1.3025	1.1354	1.0737	0.9367	0.8763	0.8949	0.8766	0.8612
Uniform: shift	0.8611	0.8162	0.8360	0.7854	0.7787	0.7938	0.7560	0.7404	0.8137
Uniform: ratio	0.8285	0.8462	0.7789	0.7685	0.7759	0.7799	0.7401	0.7747	-
Extreme negative skew: shift	1.2952	1.0213	0.9250	0.9802	1.0610	1.0431	1.0477	1.0479	-
Extreme negative skew: ratio	1.5027	1.1288	1.1332	1.2639	1.3322	1.3808	1.3442	1.2769	-
Normal: shift	0.9601	1.0049	0.8356	0.7336	0.7850	0.7865	0.7797	0.7560	0.7516
Normal: ratio	0.8230	0.9140	0.8959	0.8114	0.7702	0.8166	0.7855	0.7961	0.7781

**Table 5:** Relative efficiency of ANCOVA and Mann-Whitney combining all sample sizes. Values less than 1 indicate greater power of ANCOVA; greater than 1 indicates superiority of Mann-Whitney.

Distribution	Correlation between baseline and post-treatment score								
	
	0.1	0.2	0.3	0.4	0.5	0.6	0.7	0.8	0.9
Moderate positive skew: shift	0.9342	0.9257	0.8707	0.8532	0.8569	0.9030	0.9447	0.9538	0.9963
Moderate positive skew: ratio	1.1560	1.1300	1.1074	1.0800	0.8170	0.8656	0.9065	0.9231	0.9720
Moderate negative skew: shift	0.9392	0.9058	0.8837	0.8444	0.8799	0.9114	0.9421	0.9593	0.9885
Moderate negative skew: ratio	1.1766	1.1946	1.1467	1.0980	0.8851	0.9319	0.9671	1.0241	1.0390
Uniform: shift	0.9492	0.9170	0.8869	0.8480	0.8586	0.8977	0.9281	0.9723	1.0021
Uniform: ratio	0.9575	0.9384	0.8862	0.8499	0.8604	0.8849	0.9213	0.9467	0.9895
Extreme negative skew: shift	1.4019	1.3718	1.3339	1.3045	0.8987	0.9425	0.9475	0.9405	0.9345
Extreme negative skew: ratio	1.6914	1.6185	1.6101	1.5835	1.0153	1.0367	1.0247	1.0344	1.0062
Normal: shift	0.9700	0.9393	0.9142	0.8445	0.8372	0.8840	0.9065	0.9485	0.9734
Normal: ratio	0.9551	0.9506	0.9126	0.8791	0.8536	0.8859	0.9072	0.9293	0.9654

Table 7 compares the power of Mann-Whitney to ANCOVA on raw and log-transformed data for the distribution with extreme asymmetry. For this distribution, the non-parametric test is generally superior, though there is no simple relationship to sample size. Again, non-parametric analysis of change scores is dramatically less efficient that use of post-treatment scores. To check these data, the methods were used on the original data (n = 185). The *p*-values for Mann-Whitney on post-treatment scores, Mann-Whitney on change scores, ANCOVA on raw scores and ANCOVA on log-transformed scores were, respectively: 0.0001, 0.672, 0.216 and 0.0003.

**Table 7:** Relative efficiency of ANCOVA and Mann-Whitney for the extreme asymmetry distribution. Values less than 1 indicate greater power of ANCOVA; greater than 1 indicates superiority of Mann-Whitney. In blank cells, the power of one or both tests was 100%.

Sample size	Post-treatment score				Change score			
	
	ANCOVA v. Mann-Whitney		log ANCOVA v. Mann-Whitney		ANCOVA v. Mann-Whitney		log ANCOVA v. Mann-Whitney	
	
	Shift	Ratio	Shift	Ratio	Shift	Ratio	Shift	Ratio
10	3.0404	4.1864	0.8862	1.1179	1.2586	1.9736	0.5567	0.539
20	1.2037	2.6045	0.8073	1.2589	0.5480	0.7372	0.3473	0.2983
30	1.1503	1.7717	0.9409	1.2707	0.3084	0.3860	0.2472	0.2701
40	1.1730	1.4786	1.0233	1.4105	0.2643	0.2772	0.2446	0.2421
60	1.1853	1.2015	1.1118	1.4062	0.2115	0.2121	0.1898	0.2586
100	1.2682	1.0648	1.1842	1.4789	0.2224	0.1753	0.2065	0.2545
200	1.2880	0.9257	1.2570	1.5437	0.2078	0.1496	0.1985	0.2544
400	1.3576	0.9089	1.3112	1.5308	0.1961	0.1358	0.1816	0.2418
800	1.4222	-	1.4116	-	0.2038	-	0.1783	0.2444

Table 8 compares the estimates of treatment effects from ANCOVA with the parameter used to specify the treatment effect. For the distributions with extreme skew, the simulations were repeated without truncation, that is, ignoring maximum and minimum scores. ANCOVA appears to be unbiased where the treatment effect is a shift. Where the treatment effect is a ratio, the estimate given by ANCOVA is effectively the shift expected by a patient with the mean baseline score. The size of the bias under ratio change does not seem to be large and could be adjusted for by incorporating a term for baseline score by treatment interaction.

**Table 8:** Ratio of ANCOVA estimate of treatment effect to true treatment effect.

Moderate positive skew: shift	0.9955
Moderate positive skew: ratio	0.9890
Moderate negative skew: shift	1.0003
Moderate negative skew: ratio	0.9823
Uniform: shift	1.0028
Uniform: ratio	1.0005
Extreme asymmetry: shift	1.0067
Extreme asymmetry: ratio	0.9122
Extreme negative skew: shift	0.9973
Extreme negative skew: ratio	1.0055

## Discussion

This study complements previous work on the relative power of parametric and non-parametric statistics by examining the common situation where an outcome is measured before and after a randomly assigned treatment. The study also appears to be novel in its incorporation of different types of treatment effect: shift and ratio.

The immediate conclusions challenge the conventional wisdom of the textbooks. There is no simple and obvious manner in which non-parametric methods becomes superior once the distribution of data shifts away from normal. It is true that under normality parametric methods are trivially more efficient. But for non-normal data, the relative power of parametric and non-parametric statistics varies from distribution to distribution and depends on whether the size of the treatment effect depends on baseline score (i.e. a ratio effect). Moreover, there is no simple relationship between relative power and sample size and no clear rationale for the frequently cited threshold of 30 – 50 patients per group indicating acceptability of parametric statistics.

In general, ANCOVA outperformed Mann-Whitney for most distributions under most circumstances. This is heartening because ANCOVA has a major advantage over any non-parametric method: it provides an estimate for the size of the difference between group, that is, an effect size. Clinicians and patients generally want to know not just whether a treatment helps, but how much it helps, so they can determine whether it is worth the time, effort, risks and expense. The CONSORT group, which issues recommendations on the reporting of randomized trials, has stated that the results of a trial should stated as "a summary of results for each group, and the estimated effect size and its precision (e.g., a 95% confidence interval)". They go on to state that "although p-values may be provided ... results should not be re ported solely as p-values" [17]. ANCOVA directly provides the effect size, which appears to be unbiased; Mann-Whitney only the *p*-value. It is true that an estimate, such as a difference between medians with associated confidence interval, can be calculated separately from the Mann-Whitney and reported alongside the *p*-value. Nonetheless, the need to use separate techniques for estimation and inference must be seen as a disadvantage. Moreover, the parametric methods are also often to be preferred because estimates using medians may have little relevance for decision making. A good example comes from health economics [18]: we want to know the difference between the mean costs of two treatments because multiplying this difference by the number of patients we expect to treat gives us the expected financial impact of choosing one treatment over the other; the difference in median costs has no practical application.

Accordingly, in apparent distinction to much of the prior methodologic literature, ANCOVA should be the method of choice for analyzing randomized trials with baseline measures. Not only does it do something essential, provide an estimate, that Mann-Whitney cannot, but it appears more powerful in most circumstances. The exception is instructive: Mann-Whitney consistently outperformed ANCOVA only for a data set with extreme skew obtained from a biomarker study. Yet with such extreme skew, the estimate provided by ANCOVA – the average reduction in the biomarker – is of questionable interpretability. Rather than conclude that treatment lead to a 1.5 point drop in Ki67, it seems more appropriate to say that 32% of patients in the treatment group had zero Ki67 at follow-up compared to 14% of controls. In other words, there appears to be a link between the power of ANCOVA and the usefulness of the estimate it provides.

It should be remembered that the relative advantage of ANCOVA is primarily restricted to analysis of randomized trials. It has been argued [19] that ANCOVA with baseline scores should not be used for non-randomized trials on the grounds where baseline scores are not expected to be equivalent. For example, in measuring how anxiety of adolescent boys and girls changes after a stimulus, use of ANCOVA would address the question: "What would be the difference in changes between boys and girls given an equivalent baseline score?". Yet we would not anticipate that baseline anxiety levels of boys and girls would be the same.

This paper has not examined lumpy or multimodal distributions [8]. Yet given that the relative power of parametric methods seems primarily affected by asymmetry – compare the normal and uniform with the skewed distributions – the results cited here should apply to such distributions. This paper also did not examine semi-parametric methods, such as ANCOVA on ranks. There is some evidence that these methods are preferable to fully parametric alternatives for skewed distributions [20] and there remains the possibility of using standard ANCOVA for obtaining estimates of treatment effects and the semi-parametric test for inference.

**Table 6:** Relative efficiency of ANCOVA and Mann-Whitney combining all sample sizes. Mann-Whitney is always analyzed using the post-treatment score. Values less than 1 indicate greater power of ANCOVA; greater than 1 indicates superiority of Mann-Whitney.

Distribution	Correlation between baseline and post-treatment score								
	
	0.1	0.2	0.3	0.4	0.5	0.6	0.7	0.8	0.9
Moderate postive skew: shift	0.9397	0.9184	0.8831	0.8433	0.8012	0.7384	0.6701	0.5627	0.4214
Moderate postive skew: ratio	1.1430	1.1415	1.1036	1.0849	1.0541	0.9759	0.9090	0.7662	0.5947
Moderate negative skew: shift	0.9293	0.9281	0.8844	0.8326	0.7973	0.7256	0.6631	0.5556	0.4077
Moderate negative skew: ratio	1.1724	1.1871	1.1453	1.1103	1.0568	0.9867	0.9004	0.7665	0.5783
Uniform: shift	0.9324	0.9136	0.8926	0.8609	0.7741	0.7341	0.6483	0.5632	0.4147
Uniform: ratio	0.9475	0.9385	0.8957	0.8497	0.8191	0.7507	0.6659	0.5723	0.4297
Extreme negative skew: shift	1.4043	1.3724	1.3504	1.2859	1.2534	1.1947	1.0992	0.9472	0.7523
Extreme negative skew: ratio	1.6803	1.6120	1.6265	1.5843	1.4941	1.4206	1.2697	1.0932	0.858
Extreme negative skew: shift. ANCOVA log transformed	0.9709	0.9600	0.9638	0.9043	0.8940	0.8633	0.8109	0.7443	0.6680
Extreme negative skew: ratio. ANCOVA log transformed	0.9502	0.9298	0.9317	0.9077	0.8662	0.8282	0.7834	0.7161	0.6408
Normal: shift	0.9712	0.9258	0.9081	0.8618	0.7841	0.7272	0.6423	0.5349	0.3896
Normal: ratio	0.9550	0.9557	0.9183	0.8692	0.8139	0.7652	0.6527	0.5427	0.4131

## Pre-publication history

The pre-publication history for this paper can be accessed here:


